# Does the Germ of Yellow Fever Have Its Permanent Host in the Stegomyia Fasciata?*Read at Hearne meeting of the Brazos Valley Medical Society, January 14, 1904.

**Published:** 1904-02

**Authors:** J. P. Oliver

**Affiliations:** Caldwell, Texas


### TEXAS MEDICAL JOURNAL.

ESTABLISHED JULY, 1885.

PUBLISHED MONTHLY.—SUBSCRIPTION $1.00 A YEAR.

VOL. XIX. AUSTIN, FEBRUARY, 1904. No. 8.

### Original Contributions.

For Texas Medical Journal.

### Does the Germ of Yellow Fever Have Its Permanent Host in the Stegomyia Fasciata?*

BY J. P. OLIVER, M. D., CALDWELL, TEXAS.

The etiology of yellow fever is still a disputed point, some contending that the mosquito is the sole disseminator, and that it is the permanent host of the fever germ; while others, the writer included, believe that it may be conveyed by the mosquito by infection, or by fomites, and that the mosquito is neither the host nor the sole disseminator of the disease. The record of yellow fever in the United States furnishes abundant proof of this assertion. It has been heralded all over the world by several scientific bodies that the above mosquito is the sole disseminator of the disease. It would seem almost like utter presumption on my part to raise my voice and use my pen against such august bodies of science. But I am thankful that I live in a country where free speech is one of the bulwarks of this great American nation. I maintain that the past record of yellow fever in the United States and other places, and from the time that Columbus landed first in Cuba, does not sustain the mosquito theory of the disease, nor does the present epidemic of yellow fever, which has been prevailing in the Mexican ports and along the Texas border for the last two months sustain it.

*Read at Hearne meeting of the Brazos Valley Medical Society, January 14, 1904.

The work of Drs. Reed, Carroll, Lazear, and Aggremonte definitely proved to their own satisfaction and many other eminent men in the profession that the above mosquito was the sole disseminator of yellow fever, at the International Medical Congress in Havana in 1901. The work of Sanarelli was world wide. He found a bacillus in a large portion of his cases, which he believed was the cause of yellow fever; his experiments on some of the lower animals seem to confirm his investigations. Still other investigators failed to find the bacillus icteroides in most cases. Sternburg and his pupils, in their investigations, identified the bacillus as similar, if not identical, with the bacillus of hog cholera. Subsequent investigators as to the etiology of yellow fever from the icteroides bacillus have been discredited.

In the *Houston Post* of August 1, 1903, there was a criticism by the *Washington Post* of Surgeon General Wyman, of the Marine Hospital Service, relative to the mosquito theory of yellow fever, to which Dr. Wyman tersely replies in the following language: "In yellow fever Institute Bulletin No. 13, which is the bulletin referred to by the *Post* criticism, there can be found no statement that yellow fever is no longer a peril, nor even that the germ of yellow fever has been discovered, though it may seem evident to careful readers that there is an apparent demonstration in its life's history in the mosquito. But even this demonstration must be repeated and verified, and, moreover, a completion of the work will require a demonstration of this organism in the human being, which has not yet been done. Extra vigilence and special measures are just now being maintained by the service to prevent the conveyance of the scourge from Mexican ports to our own southern seaports, and grave anxiety will be felt from now until the appearance of frost by those who have the practical experience of yellow fever, and who are specially charged with its exclusion." Dr. Wyman has taken a very wise view of the situation of yellow fever along the Mexican ports and cities, but in spite of all extra vigilance and measures employed by the service, yellow fever has reached the Texas border towns and some of the interior towns, notably San Antonio, where twelve cases have been reported up to this writing, with a mortality of 33 1-3 per cent of the total cases. Dr. Guiteras, one of the marine experts of yellow fever and one who is thoroughly convinced that the mosquito theory of yellow fever is correct, received a telegram from Dr. Wyman October 24, 1903, "That the Pasteur Institute Commission of France has absolutely settled on the mosquito theory as the only means of spreading the disease." The lamented Pasteur himself was the greatest benefactor, perhaps, of his day to the peoples on hydrophobia, and I accept his teachings with all of may capacity along this line, but we do not know as yet, at least the writer does not, whether their investigations have demonstrated the yellow fever germ in the human blood, whether it has been confirmed by subsequent investigators. Until this is done the subject is an open one to scientific research. The report of the magnificent work of Drs. Parker, Beyer and Prothier at Vera Cruz during the summer of 1902 has been published by the Public Health and Marine Hospital Service; this report embraces a period from 1509, when Deigo De Neuces occupied the place then called New Spain, up to 1902. His forces numbered 780 men. On the first day of occupation they lost 400 men, shortly after 200 more, and at the end of fifteen months there remained only sixty survivors. From that date the fever has probably continued with variation that corresponds to the supply of immunes. The early historical data dealt only with great epidemics and correspondingly great loss of life. During the last thirty-six years a more or less accurate system of vital statistics has been kept. It is surprising to note that during this time 7861 deaths have been ascribed to this disease occurring annually. This is perhaps the most exhaustive report ever made of yellow fever at Vera Cruz, but it compares favorably with the history of yellow fever in Havana, Cuba, up to the time of its occupation by the American forces during the Spanish-American war. And I suppose the unsanitary condition of both cities was apparent to any ordinary observer. The record of the fever at Vera Cruz embraces a period from 1509 to 1902, inclusive, by this learned biological body of men, and the mosquito had occupied very little attention until a few years ago. Their examination of the blood of yellow fever patients yielded negative results as to any parasite, all bacteriological examinations were uniformly negative. Agglutination tests were undertaken with a variety of bacteria, including the bacillus icteroides of Sanarelli, but no positive results were obtained. Having exhausted these measures of isolating germs from the blood they turned their attention to the infected mosquito and announce as a result of their work the following conclusions:

1. The bacteriological examination of the blood of cases of yellow fever during life and the blood at autopsy and other organs performed immediately after death in uncomplicated cases is negative.

2. Stegomyia fasciata, when contaminated by feeding on a case of yellow fever forty-one and one-half hours after the onset of the disease and subsequently fed on sugar and water for twenty-two days one and one-half hours can, when permitted to feed on non-immune individuals, produce a severe attack of the disease.

3. Stegomyia fasciata contaminated by feeding on a case of yellow fever and after varying periods killed, sectionized and appropriately stained, present with regularity a protozoan parasite, the myxococcidium stegomyia, that can be traced through a cycle of development from the gamete to the sporozite.

4. Stegomyia fasciata fed on the blood from a case of malarial fever or normal blood or artificially fed does not harbor the parasite indicated in third conclusion.

This is certainly a very interesting report of a biological and scientific commission of learned men and bristles with the mosquito theory. But it is not conclusive as to the etiology of yellow fever, for they have not demonstrated the parasite in the human blood, of yellow fever cases. Protozoa, as is well known to science, are the lowest order of the animal kingdom, comprising organisms which consist of simple cells or colonies of cells and which have no nervous system and no circulatory organs. While the discovery of the hemosporidia in the mosquito is extremely interesting, certain difficulties at once present themselves, when considered critically, why this parasite has never been discovered in the blood. It may be, as Finley has suggested, "that contrary to the habits of the plasmodium, the germ of the yellow fever has its permanent hosts in the mosquito, and that only certain stages in the cycle of development are found in man, but it should be remembered that hemosporidia are not beyond the power of the microscope." (*St. Louis Courier,* August number.) In the February number, 22, of the *American Medicine,* Reed, Carroll and Agramonte established the mosquito theory of yellow fever exclusive of all other means, to their own satisfaction, beyond the possibility of a doubt. They state under their own report, "That the germ of yellow fever passes through a Berkfield filter. The germ or parasite of yellow fever, if it be a germ, must be very small or very elastic, or both, to pass through a filter that is impenetrable to very small bacteria. Under conclusion eleven they state that while the mode of propagation of yellow fever has now been definitely determined, the specific cause of the disease remains to be discovered. F. N. Welter (*Journal of the Association of Military Surgeons,* October, 1903), says that persons on board of a ship anchored at some distance from shore in a yellow fever port may become primarily infected with yellow fever without having set foot on shore. The infection thus caused has been traced directly or indirectly to the taking in of stores and provisions. Secondary cases have frequently happened on board of ships at a considerable distance from shore, then continued after the ships left ports. Epidemics on board of men of war have been prevented by the timely isolation—sending on shore—of early cases. In some instances an epidemic has not developed on ships after yellow fever had appeared, even when it was impossible to send the sick away from the ship in the very beginning of the attack. First cases have occurred from the sixth to the twelfth day after leaving port. Transferring a ship's crew from an infected vessel to an uninfected vessel put sudden end to the epidemic. The sick transferred from an infected vessel do not necessarily cause new foci. Here is military circular evidence directly contradictory to the mosquito theory and sustaining the former data of the etiology of yellow fever, not only on land, but aboard men of war and vessels of commerce. James Carroll (*Journal of the Medical Association,* November 28, 1903), believes he is justified in making the following conclusions:

1. The fusiform stage of the so-called myxococcideum stygomiæ of Parker, Beyer and Pothier, 1903 (which is mentioned in the foregoing article), is not connected in any way with the transmission of yellow fever.

2. This organism appears to be not a protozoan parasite (which is one of the lowest orders of the animal kingdom), but a yeast fungus. In its fusiform stage, the only form in which it was constantly present, it shows the characteristic budding, staining affinities, and vacuolation of spore formation of a blastomycele, and is found with considerable regularity in both male and female mosquitoes that have purposely been fed on over ripe bananas, to which a pure culture of wild yeast had been added in the laboratory.

3. This organism has not hitherto been found in separated examinations of mosquitoes of the genus stegomyia that have bitten yellow fever patients in the early stages of the disease, when such insects had been fed only on blood, dry sugar and water. This statement applies also to mosquitoes that are known to have produced the disease in human beings.

Before discussing the negative side of the mosquito theory, I wish to pause a few moments to complement and commend the magnificent work done by Dr. Tabor, State Health Officer, that of the Public Marine Hospital Service, also that of the military and local physicians. Their task has been an arduous one and they have all proven themselves workmen worthy of their steel, for their unceasing labors performed in Laredo night and day during the present epidemic of yellow fever. We have only to look over to Monterey, Linares, Tampico, Nuevo Laredo and other Mexican cities, where the disease has been prevailing for the last two or three months, to observe the differences in sanitation which, after all that has been said, in my opinion, is the key that unlocks and makes possible the stamping out of any epidemic. Laredo perhaps is in the best sanitary condition condition of any city in Texas, with an average mortality of about 10 per cent, while the Mexican cities are perhaps three times this amount. To close readers of the daily papers it would seem that Dr. Tabor and all of his associates, both State and national, were committed to the mosquito theory, but the very fact of a strong local quarantine established everywhere against the infected places of yellow fever contradicts the mosquito theory, and establishes the fact that the mosquito is not the only means of dissemination. Dr. Tabor, I am gratified to say, is one of the fellows of the Brazos Valley Medical Association, and he is establishing not only a State reputation as a health officer, but national also. Yellow fever has prevailed and is prevailing as an epidemic at Linares, Tampico, Vera Cruz, Monterey and Nuevo Laredo and several other towns in Mexico for the last two or three months with a mortality of from 10 to 40 per cent., in spite of all the efforts made by the proper authorities. It reached Laredo and several towns along the border early in September with a mortality of from 2 to 5 per cent. for the first thirty days, after this the mortality gradually increased to about 12 per cent. at the end of the next thirty days, or it was about at this ratio when the freeze came about the 16th of November, and the epidemic was soon ended.

I wish to state that when the epidemic was at its height, from thirty to thirty-five cases reported daily, the mortality averaged from 10 to 12 per cent., notwithstanding the daily efforts made by the sanitary officers to stamp it out. But two freezes did more to stop the scourge than all the previous work done by the oil and fumigating brigade combined. This corresponds with all previous epidemics of which I have any data. Yellow fever is endemic at Vera Cruz, so the record shows, from 1509 to 1902, inclusive, it would seem that immunes would be plentiful for the mosquito experiments.

Scarcely a summer passes without sporadic cases, and sometimes severe epidemics like the present year, mortality having reached 40 per cent., as reported by the Hoard of Bealth. Havana used to be in the same condition until the Spanish-American war was ended, and the city placed in a thorough sanitary condition by the United States offices, since which time Havana has been clear of yellow fever. I am persuaded that a large majority of yellow fever cases in the Mexican cities are due to the unsanitary condition of these cities. My wife, with whom it has been my good pleasure to live in connubial bliss for forty years, tells me that cleanliness is the next thing to godliness, and wherever properly observed, individually or collectively, health will abound, the mosquito to the contrary notwithstanding. Present data of the cities observing strict sanitary laws show beyond a reasonable doubt that epidemics of former mortality are things of the past, therefore I firmly believe that the present epidemic of yellow fever is recrudescent from former epidemics, unsanitation and perhaps the mosquito, to some extent, but not solely. Memphis, Tennessee, had a severe epidemic in 1878; the city suffered terribly, many victims were improperly interred and the scourge broke out next season, evidently recrudescent, not to say anything about the mosquito. Of the fifty-five unacclimated physicians who exposed themselves during the epidemic at Memphis in 1878, fifty-four suffered attacks of yellow fever. Here is a singular coincidence of fifty-four physicians being bitten by the stagomyia fasciata, if the mosquito theory is correct. Of these physicians who were non immunes, fifty-four out of fifty-five had the fever; think you which is the most reasonable theory how they became infected, by the mosquito, by contagion or by fomites? It is reasonable to suppose that they were constantly with the yellow fever patients, and it does seem unreasonable that fifty-four were bitten and one escaped if the mosquito is the only means of dissemination. True, he may have been an immune, but it is not reasonable to suppose that he was. In these cases the period of incubation varied from one to twenty-five days, the average duration being ten days. These physicians all remained steadfastly to their post of duty, consequently the attack which occurred on the twenty-fifth day was postponed for that length of time, during constant exposure in a locality most intensely infected. (*Pepper's System of Medicine.*) Memphis officials, seeing the unsanitary condition of their city, went to work and placed the city of Memphis in a good sanitary condition, and not a single case is recorded there since. The mosquito was not thought of, much less oiled.

A short impartial review of several cases of yellow fever at San

Antonio is opportune. Mrs. Kurkas' case, which was diagnosed by Drs. Tabor, Murray and McDaniel to be a case of genuine yellow fever, who was taken sick on the 6th day of October and died on the 11th of October. Her mother, who lived at Hondo, nursed her daughter from Thursday, went back with the body to their home and was taken sick four days afterwards, and ran the course of all the symptoms of yellow fever, dying with black vomit on the sixth or seventh day. This is a case that Dr. Tabor went to see from Laredo. The husband of the mother was taken sick a week afterwards, and died with all the symptoms of yellow fever, this making three of one family from the same house who died. Mrs. Urbenek, living directly across the street from Mrs. Kurka, and who was nursing her before her mother came, was taken sick five days after Mrs. Kurka's death, and died with all the symptoms of yellow fever, so pronounced by her attending physician, Dr. Braunegal. A boy by the name of Adams, 16 years old, living one block from Mrs. Kurka, died about one week after her death, and there was black vomit all over the bed clothes, as stated by the neighbors. A girl that lived at West End, and came by Mrs. Kurka's every morning to her work in town, was taken sick and died about ten days after Mrs. Kurka's death at the city hospital. A post mortem was made of this case and showed every change that occurs from yellow fever, making it out absolutely without any doubt a genuine case. If the mosquito infected the above cases, by what route did he come ("Red Back"), as it requires from twenty to thirty days for the mosquito to act and the incubation in these cases was not over ten?

Major Charles F. Mason, Chief Medical Officer of the Department of Texas, who was ordered to Laredo by the War Department to investigate the yellow fever situation at Laredo, Texas, in the interest of the military department, says: "My investigations through the physicians who held the autopsy on Dr. Ruiz in Nuevo Laredo, leave no doubt in my mind that the gentleman died with yellow fever. I am advised that Nuevo Laredo has two other cases, one now convalescent and another, a young lady, ill in bed. I could not go to Nuevo Laredo to make a personal investigation, as the American quarantine is so strict that even the health officers can not go across the river and return. There are a nmber of suspicious cases in Nuevo Laredo and these are said to be dengue fever, but the physicians certain of their diagnoses of the cases positively pronounce them yellow fever. The quarantine at Laredo is most rigid, the military having a perfect guard on the Fort

McIntosh front, while the Marine Hospital Service and the State have a patrol up stream forty-five miles and forty-five miles down stream, making a close guard along ninety miles of river front, yet they could not quarantine the mosquito. Laredo," says Dr. Mason, "is cleaning up thoroughly and is rapidly getting into a first class sanitary condition. The military post along the Rio Grande have everything in their jurisdiction well in hand for any emergency." Here was a strong guard for ninety miles under military rule, it seems to me that the very fact of this rigid quarantine excludes the possibility of the mosquito being the only disseminator, but that the officers in charge do not show their faith by their works. This report was on the 18th of September, last, when only three or four cases were developed. When Dr. Brumby, Houston's health officer, visited Nuevo Laredo about October 16th, he stated the number of cases of yellow fever had been greatly underestimated up to his visit; that at least 1500 cases reported dengue fever, but in reality were yellow fever with what mortality the data does not show. Recently the Governor of the State in which Nuevo Laredo is situated, stated that he believed 3000 people had been sick of yellow fever in Nuevo Laredo.

Dr. Mason says: "It has not been established when or through whom yellow fever was brought to Nuevo Laredo. It is believed that the infection was brought there by some previous arrival, whose identity is not known." September 28th Dr. Guiteras urged fighting the mosquito as the only means of stamping out the disease. He said that his theory of communication of the disease was accepted by the International Congress at Havana in 1901. Although there were many learned physicians who do not accept this theory, he is firmly convinced of its correctness. The same issue Dr. Tabor said, "he believed the fever could be transmitted by infection and that, while the mosquito undoubtedly conveyed the disease, he thought it could be acquired by infection as well." Dr. B. H. Carlton, in charge of the State Quarantine Department at the mouth of the Brazos river, in an interview on October 16th, stated that there was no doubt in his mind that the mosquito disseminated the disease, but you must remember that it is malignantly contagious, and is oftentimes carried from one point to another in a simple way. At a time like this I consider it impossible for the fever to get in the State through any of our seaports, as our system of quarantine is too complete and thorough. Every vessel that comes into port is boarded and disinfected. There is no comparison between the quarantine along the sea coast and along the Rio Grande, where individuals can slip across the river, maybe at night miles away from town, and succeed sometimes in evading them. Dr. Carlton sustains the views of Dr. Souchon of New Orleans, and many other eminent men, who do not believe that the mosquito is the only disseminator of the disease. The record of yellow fever during the last century proves this beyond the shadow of a reasonable doubt, to my mind, not to say anything about the present epidemic; but in this, the beginning of the twentieth century, when everything is pushed and rushed apparently with electric speed, commercial clubs, boards of trade, corporations of every character, are pushing the almighty dollar to the uttermost parts of the earth, the public health sinks in insignificance compared with the dollar, and the day is not far distant, in my opinion, when the political head of any medical man may fall if he raises his voice against certain theories. I maintain that the public health should be above everything else, commercialism to the contrary notwithstanding. What is life to a multimillionaire if he is deprived of health? Only retrospect the past of your lives, and how many wanderers have you seen seeking health? The mosquito theory, if correct, does away with the quarantine, for this is its ultimate aim, and I believe this to be the battle ground of the future theory of the mosquito. It seems to me that the fight against the mosquito is along this line and that of sanitation. Dr. Guiteras said he had oiled, or that the mosquito force had oiled, about 2000 barrels of water in Laredo, and the lamented Dr. Murray, of the Marine Hospital Service, said 2500 barrels of water had been oiled, and this had slain 18,000,000 of mosquitoes, which would have preyed upon the citizens of Laredo if this had not been done. The mortality of Laredo, compared to the Mexican cities, tells the story in no uncertain way that sanitation is the chief factor in the case. If the mosquito theory is correct, and they are the sole transmitters of the disease, and is the immediate host of yellow fever, there need be no stoppage of commerce on sea or land. Lock your doors and come and go through every infected port, so you don't let the mosquito puncture you with his bill; if you do, he is said to be a depositor as well as a sucker. The truth, the whole truth, and nothing but the truth is what the practical and scientific world wants. If the mosquito is the permanent host of yellow fever, surely the microscope will verify it. It is an old saying, but nevertheless true, that no fox is so crafty but what some hunter can put his hide on a pole. So it is with the germ of yellow fever, if a germ it is. Some scientist or practical medical man will establish the fact to the medical world.

Caldwell, Texas, November 9, 1904.

